# Multivitamin supplementation improves haematologic status in children born to HIV-positive women in Tanzania

**DOI:** 10.7448/IAS.16.1.18022

**Published:** 2013-08-13

**Authors:** Enju Liu, Christopher Duggan, Karim P Manji, Roland Kupka, Said Aboud, Ronald J Bosch, Rodrick R Kisenge, James Okuma, Wafaie W Fawzi

**Affiliations:** 1 Department of Global Health and Population, Harvard School of Public Health, Boston, MA, USA; 2 Division of Gastroenterology and Nutrition, Boston Children's Hospital, Boston, MA, USA; 3 Department of Pediatrics and Child Health, Muhimbili University of Health and Allied Sciences, Dar es Salaam, Tanzania; 4 United Nations Children's Fund, Regional Office for West and Central Africa, Dakar, Senegal; 5 Department of Microbiology and Immunology, Muhimbili University of Health and Allied Sciences, Dar es Salaam, Tanzania; 6 Department of Biostatistics, Harvard School of Public Health, Boston, MA, USA; 7 Department of Nutrition, Harvard School of Public Health, Boston, MA, USA; 8 Department of Epidemiology, Harvard School of Public Health, Boston, MA, USA

**Keywords:** multivitamins, haemoglobin, anaemia, mother-to-child transmission, randomized controlled trial, Tanzania

## Abstract

**Introduction:**

Anaemia is prevalent among children born to HIV-positive women, and it is associated with adverse effects on cognitive and motor development, growth, and increased risks of morbidity and mortality.

**Objective:**

To examine the effect of daily multivitamin supplementation on haematologic status and mother-to-child transmission (MTCT) of HIV through breastfeeding.

**Methods:**

A total of 2387 infants born to HIV-positive women from Dar es Salaam, Tanzania were enrolled in a randomized, double-blind, placebo-controlled trial, and provided a daily oral supplement of multivitamins (vitamin B complex, C and E) or placebo at age 6 weeks for 24 months. Among them, 2008 infants provided blood samples and had haemoglobin concentrations measured at baseline and during a follow-up period. Anaemia was defined as haemoglobin concentrations<11 g/dL and severe anaemia<8.5 g/dL.

**Results:**

Haemoglobin concentrations among children in the treatment group were significantly higher than those in the placebo group at 12 (9.77 vs. 9.64 g/dL, *p=*0.03), 18 (9.76 vs. 9.57 g/dL, *p=*0.004), and 24 months (9.93 vs. 9.75 g/dL, *p=*0.02) of follow-up. Compared to those in the placebo group, children in the treatment group had a 12% lower risk of anaemia (hazard ratio (HR): 0.88; 95% CI: 0.79–0.99; *p=*0.03). The treatment was associated with a 28% reduced risk of severe anaemia among children born to women without anaemia (HR: 0.72; 95% CI: 0.56–0.92; *p=*0.008), but not among those born to women with anaemia (HR: 1.10; 95% CI: 0.79–1.54; *p=*0.57; *p* for interaction=0.007). One thousand seven hundred fifty three infants who tested HIV-negative at baseline and had HIV testing during follow-up were included in the analysis for MTCT of HIV. No association was found between multivitamin supplements and MTCT of HIV.

**Conclusions:**

Multivitamin supplements improve haematologic status among children born to HIV-positive women. Further trials focusing on anaemia among HIV-exposed children are warranted in the context of antiretroviral therapy.

## Introduction

Anaemia is the most prevalent nutrition-related public health problem worldwide, particularly among infants and young children in resource-limited settings. Infants born to HIV-positive mothers are at higher risk for anaemia due to the direct effect of HIV on erythropoiesis [1], exposure to antiretroviral therapy (ART) [2], HIV-associated infections [3], and micronutrient deficiencies [4]. Many studies have demonstrated that supplementation of multiple micronutrients is effective to prevent or treat anaemia among young children in resource-limited countries [5–9]. However, to our knowledge, few studies have examined the effect of supplementation of micronutrients among HIV-positive or HIV-exposed children [10].

We previously observed that multiple micronutrients (B vitamins, vitamin C and vitamin E) were effective in reducing HIV progression among HIV-positive women, and the supplementation decreased the risk of mother-to-child transmission (MTCT) among immunologically and nutritionally compromised HIV-positive women through breastfeeding [11, 12]. To evaluate the efficacy of direct child supplementation on MTCT and child anaemia status, we therefore conducted a randomized trial of multivitamin supplementation (thiamine, riboflavin, niacin, vitamin B6, folate, vitamin B12, vitamin C and vitamin E) among infants born to HIV-positive women living in Dar es Salaam, Tanzania. We aimed to examine the effect of daily multivitamin supplementation on the child's haematologic status and MTCT of HIV through breastfeeding.

## Methods

### Study participants

From February 2004 to June 2007, pregnant women aged 18 years or older presenting for prenatal care at the 32nd week of gestation or earlier in one of the eight clinics in Dar es Salaam, were offered HIV screening with pre- and post-test counselling. HIV-positive women were followed up to enrol their infants into the randomized clinical trial. Infant eligibility criteria included born to HIV-positive women, singleton birth, age 5–7 weeks, and the mother intending to reside in Dar es Salaam for at least two years after delivery. We excluded from the study twin infants and infants with multiple or serious congenital anomalies (such as cyanotic congenital heart disease, spinal bifida) or other medical conditions that would interfere with their ability to comply with the study procedures. Eligible singleton birth infants were randomly assigned to receive a daily oral dose of multivitamins or placebo at age of six weeks (baseline) for 24 months. A randomization list from 1 to 2400 was prepared according to a computer-generated sequence in a block of 20. The randomization list was provided to the pharmacy department, with each a number corresponding to a code denoting one of the two treatments. Infants enrolled at the study clinic were provided the next consecutive number in the series when they received the supply of daily regimen from the pharmacist.

From age six weeks to six months, children in the multivitamin treatment group received one capsule containing 60-mg vitamin C, 8-mg vitamin E, 0.5-mg thiamine, 0.6-mg riboflavin, 4-mg niacin, 0.6 mg vitamin B6, 130-µg folate and 1-mg vitamin B12. For children older than six months, two capsules were administered daily. These doses represent 150–600% and 200–400% of US adequate intake for children aged 0–6 and 7–12 months, respectively, and 133–800% of the US Recommended Dietary Allowance (RDA) for children aged 1–3 years. For these nutrients, tolerable upper intake levels have not been defined for children<12 months; the tolerable upper intake levels (if defined) were not exceeded for children≥12 months [13–15]. We did not include vitamin A in our treatment regimen because vitamin A supplementation was associated with increased risk of MTCT through breastfeeding [11, 16]. In addition, all children in this study received periodic large doses of vitamin A supplements (100,000 IU at nine months and 200,000 IU at 15 and 21 months) as per Tanzania Ministry of Health guidelines. The multivitamin supplement used was powder encapsulated in an opaque gelatinous capsule, which was manufactured by Nutriset (Malaunay, France). Mothers were instructed on how to push the capsule through the back of the blister pack, open it, and decant the powder into a small plastic cup. Sterile water (5 mL) supplied with the supplement was added to the powder, and the dose was given to the infants orally. A pilot phase of open-label vitamin use in 12 infants and mothers confirmed that this supplement preparation and use was well-accepted by the mothers and infants. Both the placebo and active capsules contained an orange tasting powder that allowed for identical taste and appearance. All study personnel and participants were blinded to treatment assignment for the duration of the study. Only the study statisticians and the data-monitoring committee saw unblinded data, but none had any contact with study participants.

Mothers and their children were asked to return to the clinic every month for research visits and standard clinical care. Mothers were counselled on the risks and benefits of exclusive breastfeeding in keeping with WHO recommendations in place during the study period. At each clinic visit, women were asked about infant feeding in the past seven days. Information on the infant feeding during the first week of life was requested at the first post-partum visit. During monthly follow-up, infant-feeding practices (breastfeeding status and frequency, introduction of other liquid or foods including water, tea, juice, cow's milk, infant formula, porridge, mashed vegetable, meats, rice) was collected. We defined exclusive breastfeeding as feeding a child with breast milk only without additional foods. The duration of exclusive breastfeeding was calculated as the mean of infant ages at which the last time the mother reported that the child was still exclusively breastfeeding and the first time the mother reported that the child was given other foods in addition to breast milk. Compliance with the daily regimen was measured by pill counting by research nurses of unused regimen.

When the study began in 2004, routine medical care proposed for pregnant women with HIV infection included malaria prophylaxis, diagnosis and treatment for sexually transmitted diseases and prophylaxis, diagnosis and treatment of opportunistic infections, and iron and folate supplements during pregnancy. One dose of nevirapine was given to the mother at the onset of labour and another dose given to the infant within 72 hours of birth for the purpose of prevention of MTCT [17]. As the study progressed, beginning in July 2005, the availability of antiretroviral drugs increased substantially through programmes including the President's Plan for AIDS Relief (PEPFAR) and other governmental and non-governmental programmes. Women and children in the study were screened for ART eligibility and treated according to Tanzanian Ministry of Health guidelines. Based on the previous findings on the beneficial effects of vitamin B complex, C and E for pregnancy outcomes and to slow HIV disease progression [12], all women received multivitamin supplementation (vitamin B complex, C and E) during and after pregnancy.

### Laboratory measurement

Maternal HIV-1 serostatus was determined by two sequential enzyme-linked immunosorbent assay (ELISA) using Murex HIV antigen/antibody (Abbott Murex, UK) followed by the Enzygnost anti-HIV-1/2 Plus (Dade Behring, Marburg, Germany); discordant results were resolved by a Western blot test (Bio-Rad Laboratories, Hertfordshire, UK). All children were tested for HIV infection at baseline using the Amplicor HIV-1 DNA assay version 1.5 (Roche Molecular Systems, Inc., Branchburg, NJ, USA) and then again at 18 months using HIV ELISAs. Samples from children who tested positive at 18 months were then backtested using the Amplicor HIV-1 DNA assay version 1.5 to estimate the time of transmission. Children who were HIV-negative at 18 months and still breastfed were tested again before they were discharged from the study after 24 months of follow-up.

Blood specimens were requested from each mother at baseline to measure complete blood counts including haemoglobin concentrations and T-cell subset counts. Complete blood counts, haemoglobin concentrations, and T-cell subset counts were also measured for children at baseline and every six months thereafter, until the end of the follow-up. Haemoglobin concentrations were measured using AcT5 Diff AL haematology analyzer (Beckman Coulter, Jersey City, NJ, USA) and T-cell subset, for example absolute CD4+cell count and percentage were performed with the FACSCalibur system (Becton-Dickinson, San Jose, CA, USA).

### Data management and analysis

The primary outcomes of the randomized controlled trial were mortality and morbidity, and the main results have been reported [13]. Assuming a mortality rate of 12.5% in the placebo group, we planned to enrol 2360 infants to detect a 30% reduction in mortality in the treatment group with 80% power at a significant level of 0.05 [13]. Assuming haemoglobin concentrations are measured every six months, the current analysis provided 80% power to detect a difference of 0.06 g/dL between the treatment and placebo groups after 24 months of follow-up. Assuming 10% infants are HIV-positive at baseline, the data provided 80% power to detect a maximum relative risk of 0.6 for MTCT of HIV during the follow-up period. Data were double-entered and validated using Microsoft Access software. The final data sets were converted into SAS software and uploaded to a UNIX-based server in Boston, MA.

**Figure 1:**
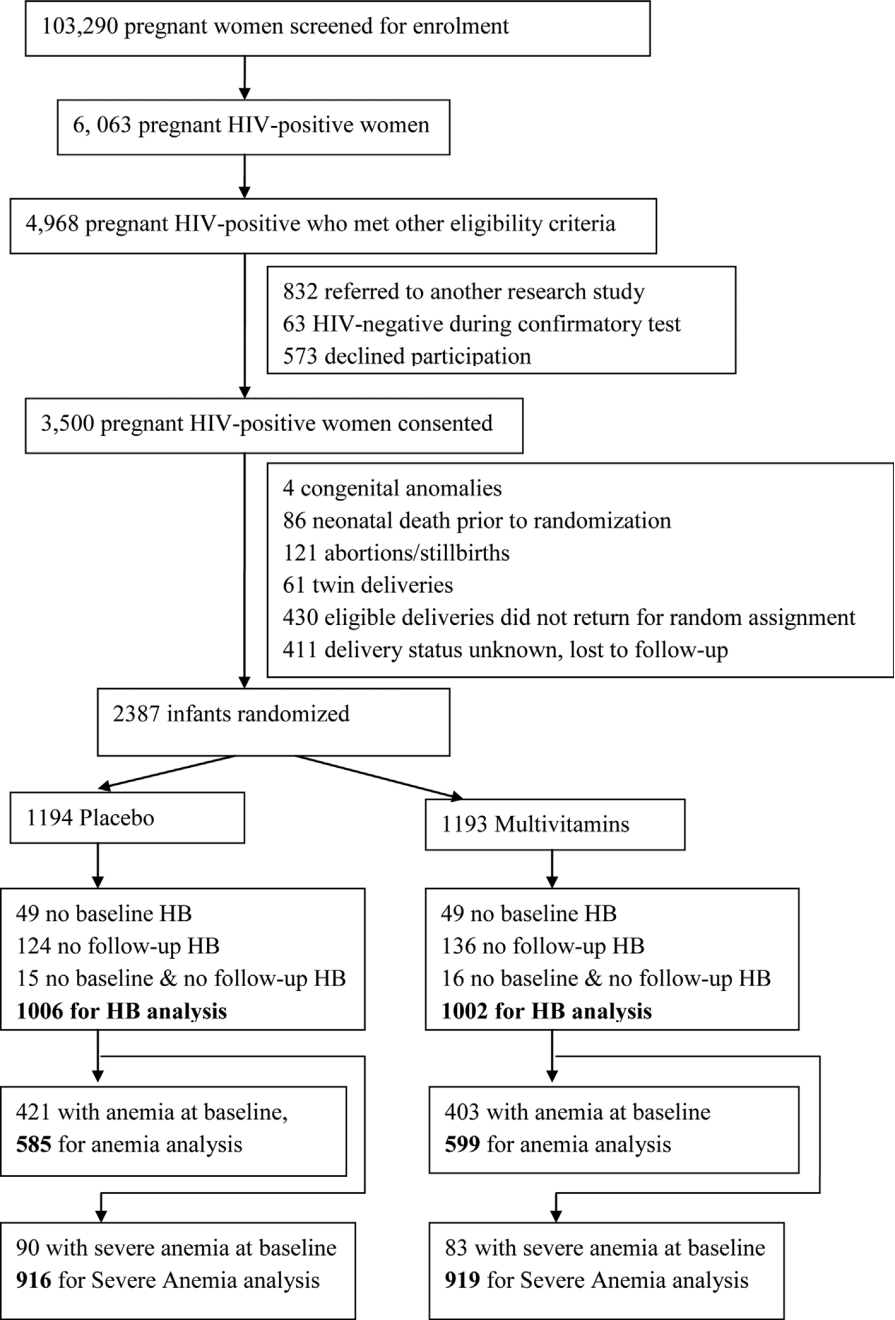
Sample sizes for analyses of haemoglobin (HB), anaemia (HB<11 g/dL), and severe anaemia (HB<8.5 g/dL).

In this article, we aimed to analyze the secondary outcomes including MTCT of HIV, child haemoglobin concentrations, and development of anaemia. Logistic regression model was used to examine the association between multivitamin supplementation and the risk of MTCT through breastfeeding. Children who tested HIV-negative at baseline and who had HIV testing during follow-up were included in the analysis for MTCT (*n*=1753). *t*-Tests were used to evaluate the effect of multivitamin supplements on haemoglobin concentrations. Cox proportional hazard models were used to examine the effect of the multivitamin supplements on the risk of development of anaemia and severe anaemia during follow-up. Children with haemoglobin measured at baseline and at least one follow-up haemoglobin measure were included in the analysis for haemoglobin concentrations (*n*=2008) (Figure 1). We further excluded children who had anaemia or severe anaemia at baseline from the respective analyses conducted to determine the risk of developing anaemia and severe anaemia during the follow-up period. Baseline measures were those obtained within six weeks after randomization. Anaemia was defined as haemoglobin levels<10.0 g/dL at baseline (six weeks of age) and<11 g/dL during follow-up, and severe anaemia was defined as haemoglobin<8.5 g/dL [18]. We classified anaemia as microcytic, normocytic, or macrocytic using mean corpuscular volume (MCV). The normal range of MCV was defined as 70–86 fL for children at the age of 6–24 months [18]. In the multivariate analyses, we considered potential confounders and independent risk factors for anaemia from a list of candidate variables. Covariates including maternal age (≤28, >28 years), CD4+ counts (<200, 200– <350, ≥350 cells/mm^3^), WHO HIV disease stage (I/II, III/IV), antiretroviral therapy during pregnancy (yes/no), sex of child (male/female), birth weight (<2.5 kg, ≥2.5 kg), preterm birth (<37, ≥37 weeks), HIV status (negative/positive), were adjusted for in the models. Likelihood ratio tests were used to test the interaction between multivitamin supplements and potential effect modifiers.

All analyses were performed using the SAS software Version 9.1 (SAS Institute, Cary, NC, USA). The significance tests were two-sided and a *p*-value less than 0.05 was considered statistically significant.

### Ethics

The Harvard School of Public Health Human Subjects Committee, and the Muhimbili University of Health and Allied Sciences Research and Publications Committee granted institutional review board approval. Written informed consent was obtained from women for HIV testing and their infant's participation in the trial. The trial was registered at clinicaltrials.gov (identifier NCT00197730).

## Results

From August 2004 until November 2007, a total of 2387 infants were randomized into placebo or multivitamin treatment groups. The two groups were comparable with respect to mother's age, education, economic status, gestational age at enrolment, CD4+ cell counts, ART during pregnancy and infant sex, birth weight, baseline weight, HIV status and CD4+ cell percentage (Table 1). Infant exclusive breastfeeding practices were not significantly different between the treatment and placebo groups. Median (25th, 75th%) regimen compliance among children was 96 (91, 99)% of the allocated regimen.

**Table 1:** Maternal and child baseline characteristics

	Placebo	Multivitamin
	
	(*N*=1006)	(*N*=1002)
Maternal characteristic		
Age (y)	28.4±5.0^a^	28.3±5.0
Education (%)		
<8 y	80	78
≥8 y	20	22
Employment (%)		
Housewife without income	65	68
Housewife with income	16	13
Others	19	19
Possessions (%)		
Have a sofa	74	72
Have a television	41	41
Have a radio	79	79
Have a refrigerator	24	25
Daily food expenditure per person (%)		
≤500 T Shillings^b^	50	54
>500 T Shillings	50	46
Marital status (%)		
Single	13	13
Married/living with partner	87	87
Prior pregnancies (%)		
0	22	22
1–3	70	70
>3	8	8
Gestational age at enrolment (wk)	24.5±5.6	24.1±5.5
Body mass index (%)		
<18.5 kg/m^2^	4	4
18.5 – <25 kg/m^2^	55	57
25.0 – <30 kg/m^2^	31	30
≥30 kg/m^2^	10	9
Haemoglobin (%)		
<11 g/dL	30	30
≥11 g/dL	70	70
CD4+ count, cells/mm^3^ (%)		
<200 cells/mm^3^	9	9
200 – <350 cells/mm^3^	18	22
≥ 350 cells/mm^3^	73	68
WHO disease stage (%)		
I/II	87	89
III/IV	13	11
On anti-retroviral therapy during pregnancy (%)	20	21
Child characteristics		
Male (%)	53	54
Birth weight (kg)	3.1±0.5	3.1±0.5
Low birth weight <2.5 kg (%)	6	6
Prematurity, <37 weeks, (%)	15	14
Weight at baseline (kg)	4.5±0.7	4.5±0.7
Length/height-for-age *z*-score at baseline	−0.4 (1.3)	−0.3 (1.3)
Weight-for-length/height *z*-score at baseline	−0.2 (1.3)	−0.1 (1.2)
Weight-for-age z-score at baseline	−0.5 (1.1)	−0.4 (1.1)
Haemoglobin at baseline (g/dL)	10.4 (1.6)	10.4 (1.5)
Haemoglobin<10 g/dL (%)	42	40
HIV positive at baseline (%)	9	9
Received ART during study period (%)	5	5
CD4+ percentage at baseline	37.3 (10.4)	37.5 (11.1)
Duration of exclusive breastfeeding (months)	3.5 (2.1)	3.7 (2.1)

a Mean±standard deviation (all such values).

b 1250 T Shillings≈ 1 USD.

New cases of HIV infection during follow-up were identified in 34/880 (3.9%) children in the placebo group and 41/873 (4.7%) in the multivitamin treatment group. There was no effect of multivitamin supplements on MTCT (odds ratio=1.23, 95% CI: 0.77–1.95, *p=*0.39). There was also no evidence of a differential effect of the supplements when testing for interactions by baseline maternal CD4+ cell counts, haemoglobin levels or sex of the child.

Haemoglobin concentrations were similar between the placebo and multivitamin treatment groups at baseline. Haemoglobin concentrations, however, were significantly higher in the treatment group at 12 months (mean 9.77 vs. 9.64 g/dL, *p=*0.03), 18 months (9.76 vs. 9.57 g/dL, *p=*0.004) and 24 months (9.93 vs. 9.75 g/dL, *p=*0.02) (Table 2). We also conducted stratified analysis by baseline HIV status, sex of the child and maternal haemoglobin levels, and found that the beneficial effect of multivitamin supplement on haemoglobin concentrations was statistically significant in HIV-negative children and girls. We did not find significant results among HIV-positive children and children born to mothers with anaemia possibly due to the small sample in these sub-groups. Tests for interaction by baseline HIV status (*p* for interaction=0.08), sex of the children (*p* for interaction=0.07), and maternal haemoglobin levels (*p* for interaction=0.80) were not statistically significant.

**Table 2:** Effects of multivitamin supplement on haemoglobin concentrations

	Placebo	Multivitamin	*P* a
Overall			
*n*	1006^b^	1002	
Baseline	10.36 (1.65)^c^	10.35 (1.47)	0.92
6 mo	9.72 (1.02)	9.71 (1.08)	0.76
12 mo	9.64 (1.23)	9.77 (1.17)	0.03
18 mo	9.57 (1.38)	9.76 (1.21)	0.004
24 mo	9.75 (1.31)	9.93 (1.35)	0.02
HIV-negative at baseline			
*n*	911	909	
Baseline	10.37 (1.66)	10.37 (1.48)	0.98
6 mo	9.78 (0.99)	9.78 (1.05)	0.96
12 mo	9.69 (1.22)	9.80 (1.14)	0.06
18 mo	9.61 (1.32)	9.80 (1.21)	0.008
24 mo	9.76 (1.32)	9.98 (1.33)	0.008
HIV positive at baseline			
*n*	88	85	
Baseline	10.39 (1.56)	10.18 (1.42)	0.35
6 mo	9.15 (1.18)	8.89 (1.08)	0.14
12 mo	9.20 (1.26)	9.35 (1.48)	0.55
18 mo	9.12 (1.97)	9.22 (1.17)	0.75
24 mo	9.62 (1.15)	9.28 (1.43)	0.26
Maternal haemoglobin concentrations ≥11 g/dL at baseline			
*n*	629	627	
Baseline	10.41 (1.65)	10.43 (1.46)	0.84
6 mo	9.76 (1.02)	9.83 (1.07)	0.27
12 mo	9.72 (1.21)	9.84 (1.17)	0.11
18 mo	9.66 (1.45)	9.81 (1.24)	0.10
24 mo	9.82 (1.25)	10.00 (1.42)	0.07
Maternal haemoglobin concentrations <11 g/dL at baseline			
*n*	272	261	
Baseline	10.24 (1.65)	10.23 (1.54)	0.92
6 mo	9.62 (1.01)	9.43 (1.12)	0.04
12 mo	9.46 (1.19)	9.61 (1.19)	0.20
18 mo	9.43 (1.26)	9.63 (1.20)	0.11
24 mo	9.56 (1.39)	9.76 (1.28)	0.21
Boys			
*n*	530	546	
Baseline	10.19 (1.49)	10.24 (1.40)	0.56
6 mo	9.64 (1.02)	9.64 (1.03)	0.96
12 mo	9.55 (1.29)	9.70 (1.20)	0.07
18 mo	9.50 (1.35)	9.63 (1.20)	0.16
24 mo	9.74 (1.31)	9.80 (1.31)	0.64
Girls			
*n*	476	456	
Baseline	10.55 (1.79)	10.49 (1.40)	0.54
6 mo	9.81 (1.02)	9.79 (1.13)	0.78
12 mo	9.74 (1.16)	9.85 (1.12)	0.15
18 mo	9.64 (1.41)	9.92 (1.22)	0.006
24 mo	9.74 (1.31)	10.08 (1.38)	0.004

a *p*-Value based on *t*-test comparing multivitamin versus placebo group.

b *n* is the number of children included in the analysis.

c Mean (standard deviation), all such values.

We examined the efficacy of multivitamin supplementation on the risk of anaemia and severe anaemia. Eight hundred and twenty four children (41%) with anaemia and 173 children (9%) with severe anaemia at baseline were excluded from the respective analyses to determine risk for anaemia and severe anaemia. During the follow-up, 1136 (96%) of the 1184 children and 458 (25%) of the 1835 children developed anaemia and severe anaemia, respectively. Compared with children in the placebo group, children in the treatment group had a 12% lower risk of developing anaemia (HR: 0.88, 95% CI: 0.79–0.99, *p=*0.03) and a 21% lower risk of developing severe anaemia (HR: 0.79, 95% CI: 0.65–0.95, *p=*0.01) after adjusting for potential confounders. Compared to children in the placebo group, children in the treatment group had a 17% reduced risk of developing normocytic anaemia and a 24% reduced risk of developing severe microcytic anaemia (HR: 0.76, 95% CI: 0.61–0.93, *p=*0.009) (Table 3).

**Table 3:** Effects of multivitamin supplementation on risk of anaemia and severe anaemia^a^

Outcomes	Placebo	Multivitamins	*p*
Anaemia (haemoglobin<11 g/dL)			
*n* b	585	599	
Person-months	3428	3694	
Number of cases	566	570	
HR (95% CI)^c^	1.00	0.88 (0.79–0.99)	0.04
HR (95% CI)^d^	1.00	0.88 (0.78–0.99)	0.03
Anaemia+microcytosis (haemoglobin<11 g/dL and MCV<70 fL)			
No. of Cases	261	287	
HR (95% CI)^c^	1.00	0.95 (0.81–1.13)	0.58
HR (95% CI)^d^	1.00	0.94 (0.80–1.12)	0.50
Anaemia+normocytosis (haemoglobin<11 g/dL and 70≤MCV<86 fL)			
Number of cases	295	280	
HR (95% CI)^c^	1.00	0.84 (0.71–0.99)	0.04
HR (95% CI)^d^	1.00	0.83 (0.70–0.98)	0.03
Anaemia+macrocytosis (haemoglobin<11 g/dL and 70≤MCV<86 fL)			
Number of cases	9	3	
HR (95%CI)^c^	1.00	0.29 (0.08–1.09)	0.07
HR (95%CI)^d^	1.00	–	–
Severe anaemia (haemoglobin<8.5 g/dL)			
*n* ^e^	916	919	
Person-months	13,653	14,063	
Number of cases	253	205	
HR (95% CI)^c^	1.00	0.78 (0.65–0.93)	0.007
HR (95% CI)^d^	1.00	0.79 (0.65–0.95)	0.01
Severe anaemia+microcytosis (haemoglobin<8.5 g/dL and MCV<70 fL)			
Number of cases	203	159	
HR (95% CI)^c^	1.00	0.75 (0.61–0.92)	0.007
HR (95% CI)^d^	1.00	0.76 (0.61–0.93)	0.009
Severe anaemia+normocytosis (haemoglobin<8.5g/dL and 70≤MCV<86 fL)			
Number of cases	45	44	
HR (95% CI)^c^	1.00	0.94 (0.62–1.42)	0.75
HR (95% CI)^d^	1.00	0.94 (0.62–1.43)	0.77
Anaemia+macrocytosis (haemoglobin<11 g/dL and 70≤MCV<86 fL)			
Number of cases	3	2	
HR (95% CI)^c^	1.00	0.63 (0.11–3.78)	0.61
HR (95% CI)^d^	1.00	–	–

a Hazard ratio (HR) and 95% CI were estimated from Cox regression for the contrast of the multivitamins treatment to placebo.

b *n* is the number of children without anaemia at baseline, included in the analysis of anaemia.

c Univariate analysis.

d Adjusted for maternal age (≤28, >28 years), haemoglobin level (<11 g/dL, ≥ 11g/dL), CD4+ counts (<200, 200– <350, ≥350 cells/mm^3^), WHO HIV disease stage (I/II, III/IV), antiretroviral therapy during pregnancy (yes/no), sex of child (male/female), birth weight (<2.5 kg, ≥2.5 kg), preterm birth (<37, ≥37 weeks), HIV status (negative/positive).

In the stratified analysis, we found that the effect of the supplements on severe anaemia was only observed among children born to women with haemoglobin concentrations ≥11g/dL at baseline (HR: 0.72, 95% CI: 0.56–0.92, *p=*0.008), not among children born to women with anaemia, defined as haemoglobin concentrations<11g/dL (HR: 1.10, 95% CI: 0.79–1.54 *p=*0.57; *p* for interaction=0.007) (Table 4). The protective effect of multivitamin supplements on anaemia and severe anaemia was not modified by birth weight, baseline HIV status, sex of child and maternal CD4+cell counts.

**Table 4:** Hazard ratio (HR) of severe anaemia (haemoglobin<8.5 g/dL) associated with multivitamin supplements by baseline maternal haemoglobin levels^a^

Maternal haemoglobin concentrations at baseline		Placebo	Treatment	*p* b	*p* c
<11 g/dL	*N*	244	238		0.007
	Person-months	3651	3360		
	Number of cases	70	73		
	HR (95% CI)^d^	1.00	1.10 (0.79–1.54)	0.57	
≥11 g/dL	*N*	575	576		
	Person-months	8712	9122		
	Number of cases	158	117		
	HR (95% CI)^d^	1.00	0.72 (0.56–0.92)	0.008	
Boys	*N*	473	491		
	Person-months	6892	7312		
	Number of cases	144	118		
	HR (95% CI)^d^	1.00	0.77 (0.60–0.99)	0.04	0.82
Girls	*N*	443	428		
	Person-months	6761	6751		
	Number of cases	109	87		
	HR (95% CI)^d^	1.00	0.79 (0.59–1.06)	0.11	

a HR and 95% CI were estimated from Cox regression for the contrast of the multivitamins treatment to placebo.

b *p*-Value estimated from Cox regression to compare the multivitamins group with placebo group.

c *p*-Value for the interaction between multivitamin supplementation and maternal haemoglobin concentrations.

d Adjusted for maternal age (≤28, >28 years), CD4+ counts (<200, 200–<350, ≥350 cells/mm^3^), WHO HIV disease stage (I/II, III/IV), antiretroviral therapy during pregnancy (yes/no), sex of child (male/female), birth weight (<2.5 kg, ≥2.5 kg), preterm birth (<37, ≥37 weeks), HIV status (negative/positive).

## Discussion

In this randomized, placebo-controlled clinical trial, we found that multivitamin supplementation (vitamin B complex, C and E) was significantly associated with increased haemoglobin concentrations and a decreased risk of anaemia among children born to HIV-positive mothers. In addition, among children born to mothers with haemoglobin concentrations ≥11g/dL, supplementation was associated with a reduced risk of severe anaemia, but not among children born to mothers with anaemia. The associations between multivitamin supplementation with haemoglobin levels and anaemia status were not significantly different between boys and girls. To our knowledge, this is the first trial in which multivitamin supplements were given to HIV-exposed infants as early as six weeks of age. The findings are consistent with a previous study in which a similar mix of multivitamins (vitamin B complex, C and E) was provided to HIV-positive women during pregnancy and in the postpartum period. The study showed that multivitamin supplementation significantly improved the haematologic status of both the mother and the child [4]. However, Chhagan et al. [10] recently examined the effect of multiple micronutrients on anaemia among a representative sample of HIV-negative children born to HIV-negative women, HIV-negative children born to HIV-positive women, and HIV-positive children in rural South Africa. They did not find significant differences between the treatment and control groups, perhaps due to the small sample size, high prevalence of anaemia at baseline and lower dose of the micronutrient supplements.

There are several plausible mechanisms by which vitamins included in the supplementation might have improved haemoglobin levels. First, vitamin C enhances the absorption of iron in the intestine [19]. Second, as an antioxidant, vitamin E inhibits the oxidative damage of erythrocyte membrane by free radicals, and this function is also performed and enhanced by vitamin C [20, 21]. Third, B vitamins, especially riboflavin (B2) and vitamin B6, play a role in the synthesis of haemoglobin, thereby enhancing erythropoiesis [22].

We found that multivitamin supplements reduced the risk of severe anaemia among children born to mothers with haemoglobin concentrations ≥11 g/dL, but not among children born to mothers with anaemia (*p* for interaction=0.007). In this study, children born to anaemic mothers had lower haemoglobin concentrations than those born to mothers without anaemia. Several longitudinal studies have also shown that maternal haemoglobin or iron status during pregnancy is associated positively with infant body iron at birth, and a significant predictor for incidence of anaemia during infancy [23–25]. In addition, women with lower haemoglobin concentrations are likely to have a diet with low iron content and feed their children with a similar diet to theirs. The low body iron store at birth and low iron intake among children born to women with anaemia might explain the difference in effect of multivitamin supplements on the risk of severe anaemia. Due to the small sample size for infants born to a mother with anaemia, we may have lacked the power to detect any protective effect of the multivitamin supplements in this subgroup, thereby were not able to assess the difference in the effect of multivitamin supplements between infants born to mothers with anaemia versus without anaemia.

A few randomized controlled trials have demonstrated that direct supplementation of multiple micronutrients is associated with increased haematologic status among young children [7, 10]. The International Research on Infant Supplementation Study, in which the supplements were given to infants as chewable tablets (or foodlets), have consistently shown that supplementation of multiple micronutrients improved anaemia status among infants aged 6–12 months in developing countries, including Indonesia, Peru, South Africa, and Vietnam [26–29]. Zlotkin and colleagues [9, 30, 31] found that the micronutrient Sprinkles, is effective in improving haemoglobin status and reducing the anaemia prevalence among young children. Iron was included in the treatment regimens in these trials [9, 26–31]. We did not provide iron in this study; however, children in the multivitamin treatment group still had higher haemoglobin concentrations and a lower risk of anaemia than those in the placebo group.

We did not include iron in our regimen because there are concerns over a possible deleterious effect of iron supplementation among non-anaemic and/or HIV-positive children. Iron is capable of inducing oxidative stress, as well as serving as an essential nutrient for microbial pathogens [32]. Some but not all observational studies have shown that high iron stores in HIV-positive individuals were associated with shorter survival time and higher mortality [33–35]. A large community-based, randomized, placebo-controlled trial in Pemba, Zanzibar demonstrated an increased risk of serious morbidity among children under the age of three years who were given routine daily iron supplements [32]. The prevalence of anaemia at the study baseline is 57%, and according to the data from the National survey 1.1% of the population in Zanzibar is HIV-positive [36]. Randomized clinical trials to assess the effect of iron supplements on morbidity and mortality among HIV-positive children are lacking [37].

Anaemia is an important public health problem among young children in resource-limited setting. Studies have repeatedly demonstrated that children with lower concentrations of haemoglobin have a high risk of mortality and morbidity [38–40]. While the observed effect of increasing haemoglobin by 0.2 g/dL appears modest at the level of an individual child, shifting the population distribution of haemoglobin by this much is likely to have a significant effect on child health and survival. All mothers in this trial were provided high-dose micronutrient supplements throughout the study period. Maternal micronutrient supplementation may positively impact quality of life among pregnant HIV-positive women and improve their breast milk nutrient quantity [41], thereby indirectly increasing infant micronutrient intake. It is possible that in areas of the world where maternal micronutrient supplementation is not a standard of care, direct infant micronutrient supplementation could result in even greater improvements in the haemoglobin status of children born to HIV-positive mothers.

We did not find any association between multivitamin supplements and risk of MTCT of HIV through breastfeeding. The result did not change in analysis stratified by sex of the child. To our knowledge, this is the first randomized study to examine whether direct multivitamin supplements administered to infants reduce MTCT of HIV. One previous study has found that maternal multivitamin supplementation (vitamin B complex, C and E) decreased the risk of MTCT of HIV among immunologically and nutritionally compromised HIV-positive women [11]. In this study, the provision of multivitamin supplementation to all women during the study period may have, in part, explained the null effect of direct multivitamin supplements to infants born to these women on MTCT of HIV.

Several limitations of the study should be noted. First, the haemoglobin and anaemia status of infants and mothers were not the primary outcomes of this randomized controlled trial, and 16% of infants without haemoglobin measures at baseline or during follow-up were not included in the analysis. Therefore, the beneficial effect of multivitamin supplementation on haemoglobin concentrations needs further examination in a large trial that focuses on haemoglobin and anaemia status in the target population. Second, in the current trial, only 20% of HIV-positive women initiated ART during pregnancy. Given the increased access to ART among HIV-positive pregnant and lactating women in resource-limited settings during the past five years, our results have to be considered carefully before being applied to other HIV-exposed child populations in the current ART era. Antiretroviral drugs, such as zidovudine and most other nucleoside reverse transcriptase inhibitors, easily cross the placenta and suppress erythroid progenitor cells [42]. A few studies have indicated that antenatal exposure to ART is associated with an increased risk of haematologic abnormalities among infants during the first three months of life [2, 43].

## Conclusion

In a randomized clinical trial, we found that multivitamin supplementation to children born to HIV-positive mothers was associated with better haematological status but not a reduction of MTCT of HIV. As iron deficiency anaemia is the most common type of anaemia among HIV-exposed or HIV-positive children, there is an urgent need for randomized control trials to examine the efficacy and safety of multivitamin supplements with iron included in the context of HIV infection and ART.
